# In-Hospital Safety in Field Conditions of Nifurtimox Eflornithine Combination Therapy (NECT) for *T. b. gambiense* Sleeping Sickness

**DOI:** 10.1371/journal.pntd.0001920

**Published:** 2012-11-29

**Authors:** Caecilia Schmid, Andrea Kuemmerle, Johannes Blum, Salah Ghabri, Victor Kande, Wilfried Mutombo, Medard Ilunga, Ismael Lumpungu, Sylvain Mutanda, Pathou Nganzobo, Digas Tete, Nono Mubwa, Mays Kisala, Severine Blesson, Olaf Valverde Mordt

**Affiliations:** 1 Department of Medicines Research, Swiss Tropical and Public Health Institute, Basel, Switzerland; 2 University of Basel, Basel, Switzerland; 3 Consultant, Paris, France; 4 Programme National de Lutte contre la Trypanosomiase Humaine Africaine (PNLTHA), Kinshasa, Democratic Republic of the Congo; 5 Bureau Diocesain d'Oeuvres Médicales (BDOM), Kikwit, Democratic Republic of the Congo; 6 DND*i*, Geneva, Switzerland; Foundation for Innovative New Diagnostics (FIND), Switzerland

## Abstract

**Background:**

*Trypanosoma brucei (T.b.) gambiense* Human African trypanosomiasis (HAT; sleeping sickness) is a fatal disease. Until 2009, available treatments for 2^nd^ stage HAT were complicated to use, expensive (eflornithine monotherapy), or toxic, and insufficiently effective in certain areas (melarsoprol). Recently, nifurtimox-eflornithine combination therapy (NECT) demonstrated good safety and efficacy in a randomised controlled trial (RCT) and was added to the World Health Organisation (WHO) essential medicines list (EML). Documentation of its safety profile in field conditions will support its wider use.

**Methodology:**

In a multicentre, open label, single arm, phase IIIb study of the use of NECT for 2^nd^ stage T.b. gambiense HAT, all patients admitted to the trial centres who fulfilled inclusion criteria were treated with NECT. The primary outcome was the proportion of patients discharged alive from hospital. Safety was further assessed based on treatment emergent adverse events (AEs) occurring during hospitalisation.

**Principal Findings:**

629 patients were treated in six HAT treatment facilities in the Democratic Republic of the Congo (DRC), including 100 children under 12, 14 pregnant and 33 breastfeeding women. The proportion of patients discharged alive after treatment completion was 98.4% (619/629; 95%CI [97.1%; 99.1%]). Of the 10 patients who died during hospitalisation, 8 presented in a bad or very bad health condition at baseline; one death was assessed as unlikely related to treatment. No major or unexpected safety concerns arose in any patient group. Most common AEs were gastro-intestinal (61%), general (46%), nervous system (mostly central; 34%) and metabolic disorders (26%). The overall safety profile was similar to previously published findings.

**Conclusions/Significance:**

In field conditions and in a wider population, including children, NECT displayed a similar tolerability profile to that described in more stringent clinical trial conditions. The in-hospital safety was comparable to published results, and long term efficacy will be confirmed after 24 months follow-up.

**Registration:**

The trial is registered at ClinicalTrials.gov, number NCT00906880.

## Author Summary

Sleeping sickness is a neglected tropical disease affecting people in Sub-Saharan Africa, most of them in poor, rural settings. If not treated, the disease usually progresses into a serious stage affecting the central nervous system, causing severe sleep disturbances, as well as other neurological and psychiatric disorders, coma, and death. Until recently, the only ways to treat 2^nd^ stage gambiense sleeping sickness involved drugs that were either toxic (such as the arsenic derivative melarsoprol) or difficult to administer in resource-constrained settings (e.g. eflornithine). A new treatment, nifurtimox-eflornithine combination therapy (NECT), was developed and tested in clinical trials, and is now recommended as the treatment of choice for 2nd stage sleeping sickness. NECT is easier to administer than eflornithine, but more information needs to be gathered for its use in the field: logistical barriers must be overcome, health care staff must be well trained, and, importantly, the safety profile of the treatment in real-life conditions needs to be better evaluated. We report here the results of a study designed to gain better understanding of the clinical tolerability, feasibility and effectiveness of NECT in field conditions, and to gather data on its use in specific populations, such as children and pregnant women.

## Introduction

Human African trypanosomiasis (HAT) is one of the most neglected tropical diseases (NTDs), suffering from a lack of optimal control tools and insufficient research efforts. It affects people in sub-Saharan Africa who often live in remote and/or insecure areas, with limited access to health care [1]. Owing to past and ongoing surveillance and control efforts of the National Control programmes from the affected countries supported mainly by WHO (World Health Organisation), BTC (Belgian Development Agency) and MSF (Médecins Sans Frontières), less than 10'000 HAT patients are currently reported each year [2]. However, funding to keep up an adequate level of control activities is nowadays increasingly difficult to obtain. Moreover, in almost all cases, HAT is fatal if untreated. *T.b. gambiense* accounts for 95% of currently reported HAT cases. The disease progresses from 1^st^ stage (infecting blood and lymph) to 2^nd^ stage (affecting the central nervous system), which ultimately leads to severe sleep disturbances, other neurological and psychiatric disorders, coma and eventually death.

Until 2009, treatment for the 2^nd^ stage of *T.b. gambiense* HAT was limited to melarsoprol, an arsenic derivative, or eflornithine. Treatment with melarsoprol is associated with high toxicity, is sometimes fatal (mean 9.4% (range 2.7–34%) [3]), and displays high rates of failure in several geographic areas [4], [5], [6], [7], [8], [9]. Although safer and more effective [4], eflornithine monotherapy comes with burdensome treatment administration requiring 56 slow infusions administered every 6 hours over 2 weeks that are difficult to implement outside well-staffed hospital settings.

A new treatment alternative, nifurtimox-eflornithine combination therapy (NECT), was added to the World Health Organisation's Essential Medicines List (WHO EML) in 2009 for the treatment of 2^nd^ stage *T.b. gambiense* HAT [10], based on its efficacy and safety profile observed in a randomised controlled trial conducted in a well-defined study population [11]. NECT is easier to administer and requires fewer hospital resources than eflornithine monotherapy, with only 14 slow infusions administered every 12 hours for 1 week, with a concurrent 10 days oral treatment with nifurtimox. The needed quantity of eflornithine for NECT (and consequently the drug production and transportation cost) is 2 times lower than for the eflornithine monotherapy regimen. NECT now stands as the preferred first-line treatment for 2^nd^ stage *T.b. gambiense* HAT. However, it is not yet the ideal HAT treatment and to enable its wider use in remote rural settings, financial and logistical barriers must be overcome, health care staff must be well trained, and, importantly, the safety profile under such conditions needs to be better known.

This study aimed to further document the clinical tolerability, feasibility and effectiveness of treatment with NECT in field conditions, *i.e.* with less stringent inclusion/exclusion criteria and in a larger population including children, pregnant and breastfeeding women and patients with a HAT treatment history.

## Methods

### Objectives

The primary objective was to assess the clinical response of NECT for the treatment of 2^nd^ stage *T.b. gambiense* HAT in field conditions. Secondary objectives included assessing the incidence and type of adverse events (AE), the feasibility of NECT implementation by the health facilities and the effectiveness of NECT at 24 months after treatment. Patient follow-up is still ongoing at the time of publication, therefore the current analysis deals with in-hospitalisation safety only.

### Outcomes

The primary outcome was the proportion of patients discharged alive from the hospital (treatment facility). This was directly assessed by the site Investigators after treatment at the time of discharge. Secondary outcomes were frequency, nature, severity and relatedness of adverse events and adherence to treatment (interruptions, cessations, dose deviations, length of hospitalisation).

### Study design, settings and participants

This was a multicentre, open label, single arm, phase IIIb study of the therapeutic use of NECT for treatment of 2^nd^ stage *T.b. gambiense* HAT in the Democratic Republic of the Congo (DRC). The study took place in two of the most endemic provinces, Bandundu and Kasai Oriental, at five HAT treatment facilities operated by the national HAT control program (Programme National de Lutte contre la Trypanosomiase Humaine Africaine, PNLTHA): Bandundu, Dipumba, Katanda, Kwamouth and Ngandajika and one treatment facility operated by the BDOM/KIKWIT (Bureau Diocesain d'Oeuvres Médicales) and supervised by the PNLTHA: General Hospital of Yasa Bonga.

All second stage HAT patients admitted to the treatment facilities and routinely diagnosed according to the national guidelines and who gave their Informed Consent for participation, were included in the trial. At inclusion, special attention was given to children and pregnant and breastfeeding women. It was under the Investigator's decision to include these sub-populations.

Exclusion criteria were inability to take oral medication and impossibility to use a nasogastric tube, treatment failure after previous NECT treatment or any other condition for which the Investigator judged that another treatment was warranted. The patients were hospitalised and treated with NECT and monitored for adverse events. During the trial, an independent Data Safety Monitoring Board (DSMB) reviewed the data for patient safety. No interim analyses were done.

### Intervention

Prior to NECT initiation, the patients received standard pre-treatment (according to the facilities' guidelines, usually antimalarial, anthelminthic and antipyretic/analgesic medication). All patients received NECT [11], the co-administration of nifurtimox (oral 15 mg/kg/day, three times a day) for 10 days and eflornithine (slow intravenous infusions, 400 mg/kg/day, twice a day) for 7 days.

All patients, including the children and offspring of pregnant or breastfeeding women, were monitored during treatment and followed-up for safety issues. Patients underwent daily evaluations, including vital signs, physical examination, adverse event monitoring, and recording of concomitant medications throughout the admission and treatment period, as well as at the end of treatment, just before patients were discharged from hospital.

The severity of the treatment emergent adverse events was graded by the Investigator, according to the Common Toxicity Criteria for adverse events (CTCAE, v03 [12] from grades 1 to 5 as mild, moderate, severe, life-threatening and death) and related to the treatment according to the Investigator's analysis. Treatment emergent adverse events were evaluated as being probably, possibly or not related to the study drugs.

Follow-up assessment visits were scheduled 6, 12, 18 and 24 months after the end of the treatment. Twenty-four months after end of treatment, patients will be assessed for the final effectiveness of treatment; these results will be reported later.

### Sample size

According to available literature [4], [11], [13], [14], the proportion of patients discharged alive from hospital (P_1_) was expected to be equal or superior to 98%. The proportion of patients discharged alive from hospital (under the null hypothesis) was assumed to be equal to P_0_ = 96%, which corresponds to the average of proportions obtained in previous studies on eflornithine.

The sample size was calculated according to a confidence interval approach with a precision of 2%. According to Fleiss' method [15], a sample size of 620 achieved 80% power to detect a difference (P_1_-P_0_) of 2% using a two-sided binomial test. The target significance level was 5%. Sample size was performed using PASS 2008 [16].

### Statistical analysis

The main analysis set was the safety population, which included all subjects who received at least one dose of study drug. For this trial design, safety and ITT (intention to treat) populations are the same. As the percentage of patients who had protocol deviations was very small (0.7%), the per-protocol population was not analysed.

All statistical evaluations were descriptive, as the aim of the study was to further document NECT implemented in field conditions and the trial was open-label and uncontrolled. Means, standard deviations and number of patients were provided for continuous variables, as well as frequency distributions for binary and categorical variables. An exact Wilson 95% confidence interval was calculated for the primary outcome. Statistical analyses were performed using the SAS software version 9.1 (SAS Institute, Cary, NC).

All data were captured at the participating treatment facility on patient case report forms (CRFs). Data were double-entered and discrepancies reviewed and corrected against the hard copy CRF.

Adverse events were coded with the MedDRA dictionary (version 11.0 [17]) and concomitant treatments with the WHO-Drug dictionary [18].

### Ethics

This research was conducted in full accordance with the ethical principles for medical research involving human subjects, as expressed in the Declaration of Helsinki and following amendments. Eligible patients were asked to meet the study Investigator or his delegate, who explained the study protocol in detail according to the patient information sheet, and requested written consent from the patient or, in case of minors, severely ill or mentally impaired patients unable to fully consent, from her/his parent(s)/guardian(s). Whenever possible (depending on age and level of understanding), the children received the information and their assent was obtained. Two Ethics Committees approved the study protocol: the Ethics Committee of both cantons of Basel (EKBB, Basel, Switzerland; 26 February, 2009) and the local Ethics Committee in the DRC (Comite d'Ethique sur la Trypanosomiase Humaine Africaine, Kinshasa, Democratic Republic of the Congo, 7 May 2009). All interventions (including follow-up visits) were free of charge to the patients.

## Results

The total enrolment period lasted 13 months and took place between May 2009 and May 2010 in six HAT treatment facilities of the DRC. One facility (Yasa Bonga) recruited patients only between November 2009 and May 2010. Here, we report the in-hospitalisation safety of 629 2^nd^ stage HAT patients treated with NECT.

### Study population - participant flow

**Figure 1:**
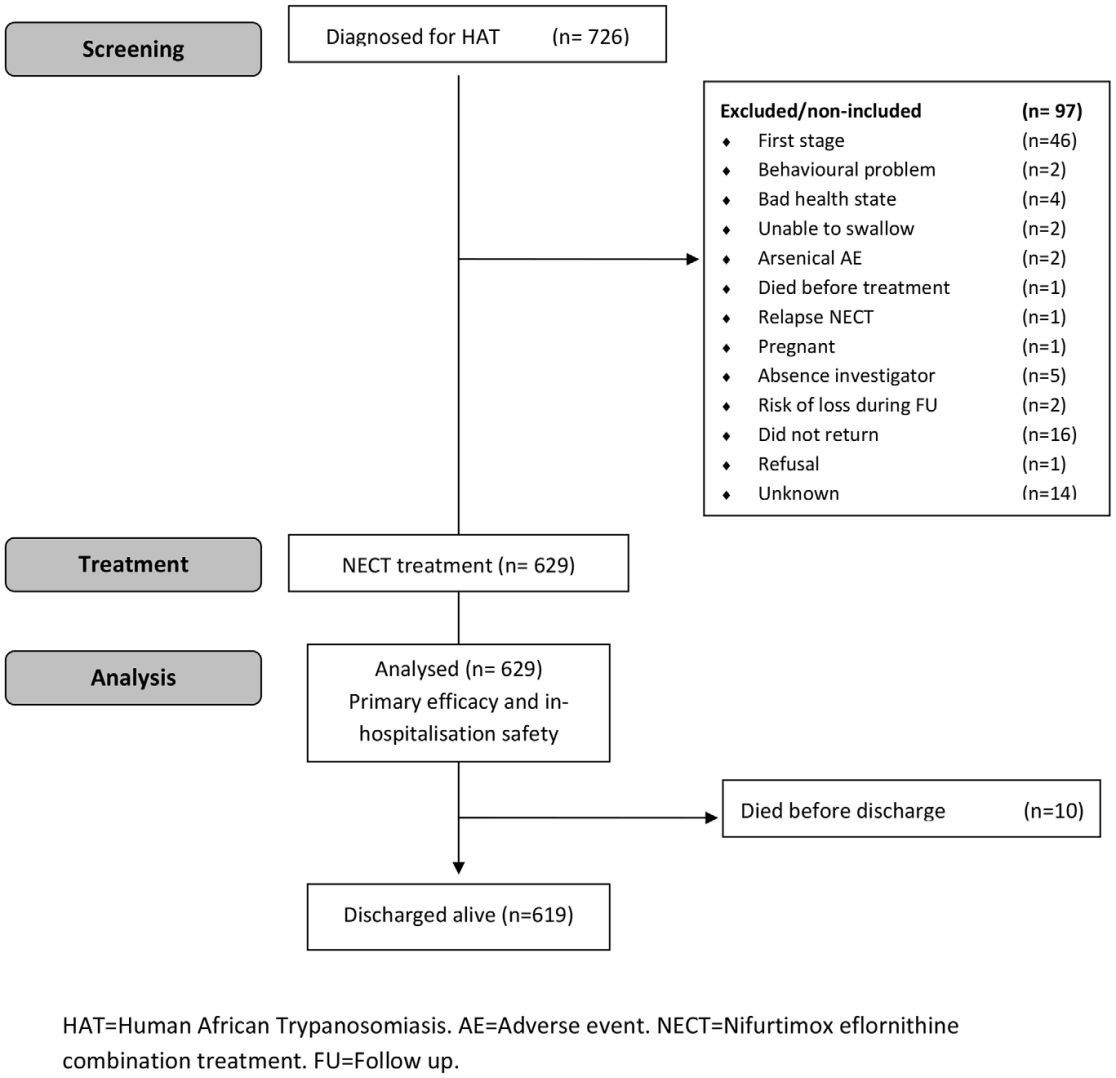
Flow diagram of the multicentre NECT trial for treatment of 2^nd^ stage HAT. The diagram includes detailed information on excluded patients.

Patients who reported passively or were sent by the mobile teams to the HAT treatment facilities were screened and diagnosed for HAT. 726 patients were diagnosed as HAT cases, of them, 680 were classified in stage 2 and potentially eligible for participation, 49 were excluded by the Investigators or failed to show up for treatment, 1 refused and 630 gave their informed consent. One patient died prior to receiving any medication and was not included in the analysis. In total, 629 patients were treated with NECT at the HAT treatment facilities. Reasons for non-inclusion and the patient flow are detailed in Figure 1.

### Baseline

The demographic, diagnostic and clinical characteristics varied between the treatment facilities (Table 1). About 16% of the patients were children below 12 years of age. Katanda had the highest (22%) rate of children admitted and Bandundu (9%) the lowest. The overall ratio of males to females was 1.3 (range 0.9 to 2.0). Amongst the patients, there were 33 (5%) breastfeeding women, 14 (2%) pregnant women and 135 (22%) patients with HAT treatment history (range 3% to 39%). Of patients with a HAT history, two thirds were considered true relapses (84/135) who were treated for HAT within 2 years prior admission to the current treatment (Table 1). There were 188 (30%) malnourished patients (172 (33%) adults and 16 (16%) children aged below 12 years,; based on age specific underweight classification of anthropometric data according to WHO [19], [20]). However, the extent of malnutrition amongst the study population varied between the facilities, with Dipumba reporting the lowest (15%) and Bandundu and Yasa Bonga the highest proportion (40%) of undernourished patients admitted.

**Table 1:** Baseline demographic, diagnostic and clinical characteristics of the patients, by centre.

Demographic characteristics	All patients	KASAI ORIENTAL province			BANDUNDU province		
		Dipumba	Katanda	Ngandajika	Bandundu	Kwamouth	Yasa Bonga
n (%) if not otherwise noted							
Number of Patients treated	629	146	132	94	98	97	62
Children 0–4 years	35 (6%)	7 (5%)	7 (5%)	5 (5%)	5 (5%)	5 (5%)	6 (10%)
Children 5–11 years	65 (10%)	15 (10%)	22 (17%)	9 (10%)	4 (4%)	8 (8%)	7 (11%)
Adolescents/Adults >11 years	529 (84%)	124 (85%)	102 (77%)	80 (85%)	89 (91%)	84 (87%)	50 (81%)
Male/female ratio	1.3	1.8	1.2	2.0	0.9	0.9	1.1
Breastfeeding women	33 (5%)	9 (6%)	2 (2%)	2 (2%)	7 (7%)	9 (9%)	4 (6%)
Pregnant women	14 (2%)	1 (1%)	4 (3%)	1 (1%)	3 (3%)	2 (2%)	3 (5%)
Age, years, mean (SD)*	30 (16)	32 (16)	26 (15)	27 (14)	35 (18)	29 (16)	27 (18)
Weight, kg, mean (SD)	45 (16)	50 (17)	44 (17)	47 (18)	45 (14)	44 (14)	38 (16)
Malnutrition by age category							
0–4 years: Weight for height >−2SD	2 (0%)	0 (0%)	0 (0%)	1 (1%)	0 (0%)	0 (0%)	1 (1%)
5–14 years: Body-mass Index for age >−2SD	14 (2%)	3 (2%)	4 (3%)	2 (2%)	1 (1%)	2 (2%)	2 (3%)
>14 years: Body-mass index <18.5 kg/m2	172 (27%)	22 (15%)	19 (14%)	29 (31%)	39 (40%)	38 (39%)	25 (40%)
Previous HAT	135 (21%)	27 (18%)	51 (39%)	24 (26%)	12 (12%)	19 (20%)	2 (3%)
within 2 years of admission	84 (13%)	18 (12%)	27 (21%)	20 (21%)	8 (8%)	9 (9%)	2 (3%)
**Parasitological findings**							
Presence of trypanosomes	558 (89%)	132 (90%)	128 (97%)	80 (85%)	85 (87%)	75 (77%)	58 (94%)
in lymph nodes	218 (35%)	53 (37%)	32 (24%)	44 (47%)	23 (23%)	40 (41%)	26 (42%)
in blood	162 (26%)	25 (17%)	32 (24%)	7 (8%)	44 (45%)	18 (19%)	36 (58%)
in CSF‡	341 (54%)	99 (68%)	86 (65%)	39 (42%)	49 (50%)	41 (42%)	27 (43%)
Leucocyte count in CSF, cells per µl, median	153	207	208	183	89	114	49
lower (25%) and upper (75%) quartiles	49; 345	96; 380	98; 442	85; 442	23; 250	25; 382	11; 172
>100 cells per µl	397 (63%)	108 (74%)	98 (74%)	66 (70%)	48 (49%)	53 (54%)	24 (39%)
**Clinical characteristics**							
Karnofski index§, mean (SD)	70 (12)	65 (15)	67 (9)	68 (10)	74 (10)	70 (8)	80 (13)
Altered or bad general health state	502 (80%)	106 (73%)	108 (82%)	88 (94%)	69 (70%)	96 (99%)	35 (56%)
Lymphadenopathy	340 (54%)	73 (50%)	61 (46%)	62 (66%)	46 (47%)	69 (71%)	30 (48%)
Facial oedema	63 (10%)	11 (8%)	21 (16%)	22 (23%)	1 (1%)	4 (4%)	2 (3%)
Fever, axillary >37.5°C	38 (6%)	13 (9%)	4 (3%)	3 (3%)	10 (10%)	0 (0%)	8 (13%)
Sleeping disorders (insomnia, somnolence)	503 (80%)	126 (86%)	117 (89%)	83 (88%)	74 (76%)	60 (62%)	40 (64%)
Headache	472 (75%)	92 (63%)	115 (87%)	69 (73%)	72 (73%)	73 (75%)	49 (79%)
Asthenia	384 (61%)	107 (73%)	115 87%)	51 (54%)	69 (70%)	24 (25%)	20 (32%)
Fever history	371 (59%)	61 (42%)	111 (84%)	40 (43%)	63 (64%)	66 (68%)	30 (48%)
Pruritus	365 (58%)	102 (70%)	104 (79%)	45 (48%)	49 (50%)	41 (42%)	24 (39%)
Weight loss	346 (55%)	61 (42%)	103 (78%)	10 (11%)	73 (74%)	84 (87%)	13 (21%)
Tremor	277 (44%)	88 (60%)	82 (62%)	36 (38%)	44 (45%)	19 (20%)	5 (8%)
Walking disorder	258 (41%)	74 (51%)	86 (65%)	39 (41%)	49 (50%)	5 (5%)	4 (6%)
Behavioral disorder	208 (33%)	35 (24%)	74 (56%)	18 (19%)	36 (37%)	32 (33%)	16 (26%)
Convulsions	31 (5%)	10 (7%)	12 (9%)	2 (2%)	3 (3%)	4 (4%)	2 (3%)

All patients were diagnosed as stage 2 HAT patients with >5 leucocytes per µl CSF (cerebro-spinal fluid), of those, 89% (558/629) were parasitologically confirmed and 11% (71/629) based on their raised WBC (white blood cell) levels in CSF and clinical signs. The latter presented either positive serology (7%, 44/629) or had already a HAT history (4%, 27/629, data not shown). The clinical and diagnostic findings differed slightly between sites.

Common disease symptoms by frequency of occurrence were sleeping disorders (nocturnal insomnia and/or diurnal somnolence), headache, asthenia, fever, pruritus and weight loss, each affecting more than half of all patients (Table 1).

### Treatment and adherence to treatment

All patients were hospitalised for the entire treatment period. Ninety-nine percent (621/629) and 94% (590/629) of the patients received the complete eflornithine and nifurtimox doses, respectively (as per protocol 14 intravenous doses of eflornithine and 30 oral doses of nifurtimox). Minor divergences occurred in the timing of drug administration or by administration of additional doses of nifurtimox (at the end of treatment) and/or in case of vomiting within the first 30 minutes after the intake. Major treatment deviations were due to overdosing of more than 10% of correct treatment (calculated based on body weight; 3 patients) or to withdrawal by family (1 patient) or to treatment cessation after 1 dose due to intolerance (1 patient). The adherence to treatment varied slightly between the facilities and also amongst the patient groups - lowest adherence was in the pregnant women group (nifurtimox 79% & eflornithine 93%) and in small children below 5 years of age (nifurtimox 89% & eflornithine 86%; Table 2).

**Table 2:** Treatment compliance, length of hospitalisation and in-hospitalisation safety of NECT by sub-population of interest.

Number of patients with	All patients							
	Total	Children		Adolescents/adults		Other female		Previous HAT
		0–4 years	5–11 years	Male	Female	Pregnant women	Breastfeeding women	within 2 years
n (%)	(N = 629)	(N = 35)	(N = 65)	(N = 299)	(N = 183)	(N = 14)	(N = 33)	(N = 84)
**Treatment and adherence**								
Treatment adherence								
30 nifurtimox doses received	590 (94%)	31 (89%)	63 (97%)	278 (93%)	177 (97%)	11 (79%)	30 (91%)	79 (94%)
14 eflornithine doses received	621 (99%)	30 (86%)	65 (100%)	298 (100%)	182 (100%)	13 (93%)	33 (100%)	82 (98%)
Patients with concomitant treatment	585 (94%)	30 (86%)	57 (88%)	281 (94%)	171 (93.4%)	14 (100%)	32 (97%)	76 (91%)
median concomitant drugs per patient	4	4	4	4	5	6	4	5
**Length of hospitalisation** *								
Median stay, in days	16	16	16	16	16	15.5	15	16
Range, in days	5–46	12–40	12–27	10–46	6–36	5–26	12–31	5–36
**In-hospitalisation safety**								
Any adverse event	578 (92%)	31 (89%)	61 (94%)	273 (91%)	171 (93%)	14 (100%)	28 (85%)	77 (92%)
mean adverse events per patient	4.2	2.7	3.4	4.3	4.5	5.7	4.2	4.5
Related adverse event†	556 (88%)	30 (86%)	57 (88%)	264 (88%)	164 (90%)	14 (100%)	27 (82%)	71 (85%)
Severe adverse event‡	79 (13%)	4 (11%)	5 (8%)	38 (13%)	26 (14%)	3 (21%)	1 (3%)	12 (14%)
Serious adverse event	32 (5%)	1 (3%)	1 (2%)	16 (5%)	10 (6%)	1 (7%)	3 (9%)	5 (6%)
possibly or probably related to treatment	25 (4%)	1 (3%)	0	12 (4%)	8 (4%)	1 (7%)	3 (9%)	4 (5%)
Death during hospitalisation	10 (1.6%)	0	0	4 (1.3%)	5 (2.7%)	1 (7%)	0	1 (1.2%)
Discharged alive	619 (98.4%)	35 100%)	65 (100%)	295 (98.7%)	178 (97.3%)	13 (92.9%)	33 (100%)	83 (98.8%)

HAT = Human African trypanosomiasis.

* Calculated from day of admission to day of discharge.

† Possibly or probably related to study drug.

‡ CTC grades 3–5.

Concomitant medication during NECT therapy was common: 93% (585/629) of patients received in median 4 additional different drugs (range 1–14). All pregnant women (14/14) and 86% (30/35) of children below 5 years of age received concomitant treatment.

The median length of hospitalisation was 16 days, and measured from the day of admission to the day of discharge, including days when pre-medication was given (Table 2). The length of the hospitalisation stay was similar for all patient groups, but varied between the facilities (12 days in Bandundu, 15 days in Dipumba, 16 days in Katanda, Ngandajika & Kwamouth, and 20 days in Yasa Bonga) mainly due to differing routine practices at the facilities (reduced pre-treatment days in Bandundu or prolonged observation days in Yasa Bonga).

### In-hospitalisation safety

98.4% (619/629) of patients were discharged alive (95%CI = [97.1%; 99.1%], Table 2). All children younger than 12 years of age were discharged alive (100/100), as were breastfeeding women (33/33). Due to a death of one pregnant woman, the survival in that group was low, at 92.9% (13/14). The proportions of patients discharged alive were similar for all other adults (97.3%–98.8%), including for patients with history of previous HAT.

At the time of discharge from the treatment facility, clinical characteristics had substantially improved (Karnofsky index, the classification of patients performance as to their functional impairment raised from mean 70% to 86%; neurological signs decreased from 89% to 37%, bad general health state had reduced from 80% to 14% and lymphadenopathy from 54% to 22%; the patients who died during the treatment were excluded from this analysis; data not shown).

Treatment emergent adverse events were reported from the first dose of study drug until discharge of the patient from the treatment facility. Five hundred and seventy-eight patients (92%) suffered from at least one adverse event and 79 patients (13%) from at least one severe adverse event (CTCAE grades 3 to 5 including death cases). The overall safety profile and the nature of the most frequently observed adverse events and severe adverse events were similar for the study populations of interest, with a few exceptions (Table 3). Pregnant women tended to be affected more frequently by vomiting, asthenia and headache. On the other hand, it was the only group not developing any psychiatric disorder. Breastfeeding women showed double the incidence of agitation than others (12%). Children had less psychiatric events and reported less headaches, but had more fever and injection site reactions. Anorexia was more frequently, and fever less frequently reported in the population with previous HAT.

**Table 3:** Incidence of treatment emergent adverse events and treatment interruptions by sub-population of interest.

Incidence of adverse events (AE in %)	All patients									Previous
	Total			Children		Adolescents/adults		Other adults		HAT
System Organ ClassLowest Level Term LLT	any AE	severe AE*	serious AE	0–4 years	5–11years	Male	Female	Breastfeeding women	Pregnant women	within 2 years
	(N = 629)			(N = 35)	(N = 65)	(N = 299)	(N = 183)	(N = 33)	(N = 14)	(N = 84)
**Patients with at least 1 adverse event**	**92**	**13.0**	**5.1**	**89**	**94**	**91**	**93**	**85**	**100**	**92**
**Gastrointestinal disorders**	**61**	**1.1**	**0.5**	**37**	**46**	**60**	**71**	**67**	**93**	**64**
Vomiting	43	0.3	0.0	14	40	39	51	58	79	49
Nausea	20	0.2	0.0	6	17	21	24	6	21	23
Colitis	7	0	0	3	8	8	8	3	0	11
Diarrhea	7	0.2	0.3	14	5	6	9	3	7	7
Epigastralgia	6	0.2	0	0	2	4	12	3	21	6
Abdominal pain	6	0.3	0	6	3	6	6	0	14	6
**General disorders and administration site conditions**	**46**	**2.9**	**0.6**	**63**	**54**	**39**	**46**	**61**	**86**	**35**
Fever	30	0.3	0	49	42	24	28	42	29	17
Asthenia	18	1.9	0	20	9	13	23	27	57	20
Injection site reaction	3	0	0	6	5	4	1	0	0	2
Other (health state, death)	0.6	0.6	0.6	0	0	0.7	0.5	0	7	1
**Nervous system disorders**	**34**	**6.0**	**2.1**	**14**	**25**	**37**	**37**	**24**	**57**	**35**
Headache	15	2.5	0	3	11	14	18	12	36	16
Dizziness	11	0.3	0	0	0	11	16	6	14	8
Convulsions	9	0.6	1.0	9	11	10	7	9	0	12
Coma	1	1	0.5	0	0	0.5	0.3	0	7	1
Ataxia	0.3	0.2	0.2	0	0	0	0.3	0	0	1
**Metabolism and nutrition disorders**	**26**	**0.2**	**0**	**17**	**25**	**29**	**26**	**12**	**29**	**38**
Anorexia	25	0	0	17	23	28	25	9	29	38
**Psychiatric disorders**	**16**	**1.7**	**0.5**	**3**	**12**	**18**	**17**	**12**	**0**	**18**
Insomnia	6	0	0	0	5	8	7	0	0	7
Agitation	6	0.8	0	3	6	5	7	12	0	5
Other (Delirium, psychose, mood disorder)	1.7	0.5	0.5	0	2	2	1	6	0	1
**Musculoskeletal and connective tissue disorders**	**14**	**0.3**	**0**	**0**	**6**	**17**	**13**	**15**	**21**	**10**
Lumbago	6	0	0	0	3	8	4	6	21	6
**Respiratory, thoracic and mediastinal disorders**	**11**	**0.6**	**0**	**9**	**6**	**13**	**8**	**9**	**14**	**10**
Cough	3	0	0	6	2	3	2	0	7	4
**Skin and sub-cutaneous tissue disorders**	**9**	**1.7**	**0**	**6**	**11**	**10**	**9**	**6**	**7**	**10**
Pruritus	7	1.7	0	6	6	7	6	6	7	6
**Vascular disorders**	**8**	**1.3**	**0.3**	**3**	**3**	**7**	**10**	**6**	**14**	**5**
Hypotension	5	0.8	0.3	3	3	3	8	6	14	4
**Cardiac disorders**	**7**	**0.3**	**0.2**	**6**	**3**	**8**	**8**	**3**	**7**	**7**
Palpitations	4	0	0	0	0	4	6	0	0	2
Cardiogenic shock	0.2	0.2	0.2	0	0	0	1	0	0	0
**Infections and infestations**	**5**	**2.7**	**1.0**	**11**	**6**	**5**	**4**	**3**	**14**	**2**
Cellulitis	1	0	0	3	0	1	1	0	7	0
**Patients with, n (%)**										
Permanent treatment interruption†	2 (0.3)			0	0	1 (0.3)	1 (0.5)	0	0	0
Repeated doses of nifurtimox	41 (6.5)			3 (8.6)	4 (6.2)	21 (7.0)	8 (4.4)	3 (9.1)	2 (14.2)	10 (11.9)

HAT = Human African Trypanosomiasis.

* CTC grades 3–5.

† Excluding treatment interruptions due to death (3 patients; 1 pregnant woman, 1 female and 1 male patient).

6.5% of the patients received a repeated or added dose of nifurtimox, often due to vomiting during the 30 minutes following drug intake. This tended to occur more frequently in small children (8.6%), pregnant (14.1%) and breastfeeding (9.1%) women and patients with previous HAT (11.9%). One patient ceased his NECT treatment due to recurrent convulsions occurring just after 1 eflornithine administration, without having had a known convulsion history. One patient had a temporary nifurtimox interruption of 16 hours due to coma.

Thirty-two (5.1%) patients had a serious adverse event (SAE) during hospitalisation. From those, 25 were considered as possibly or probably related to study drug (Table 2). Thirteen patients had an SAE affecting the nervous system including mood disorders, psychosis, convulsions, ataxia and coma, while 8 patients had an SAE that may have been induced by myelotoxicity, including infections and anaemia (data not shown). All patients with an SAE who did not die recovered without sequelae. No SAE was reported for children of breastfeeding mothers during their hospitalisation (Table 2).

During hospitalisation, 10 patients died of various causes (infections, coma, anaemia, cardiogenic shock and non-specific diagnosis). Three patients died during treatment, whilst 7 died during the observation period. Eight of the 10 patients were already in a bad health state prior to treatment, as reflected by their Karnofsky Index at 60% or lower. Nine deaths were considered to be possibly (7/9) or probably (2/9) related to NECT. One death was considered unrelated.

## Discussion

Based on the results of randomised controlled trials [11], [21] and a case series [22], NECT is currently used as first-line treatment in several African countries to treat 2^nd^ stage *T.b. gambiense* HAT. To better implement NECT in remote rural HAT treatment facilities, the knowledge on the safety profile under such conditions must be more extensively known. Here, we report the in-hospitalisation safety of NECT obtained from 629 treated HAT patients in six different treatment facilities in the DRC. Also, for the first time, special populations, such as children below 15 years of age, pregnant women and HAT relapses, were treated with NECT and are described here. The effectiveness analysis will be available once the 24 months follow-up of patients is completed in late 2012. A matter of concern may be that similar dosages as for adults have been administered to children below age 12, as in monotherapy higher doses of nifurtimox and eflornithine are recommended for children.

### NECT in-hospitalisation safety in field conditions

Almost all - 98.4% (619/629) patients were discharged alive after treatment. The nature, frequency and intensity of adverse events were situated in the expected range as shown in previous studies [11], [21], [22] and considering the fatal outcome of the disease. NECT was sufficiently tolerated among all population sub-groups in the context of 2^nd^ stage HAT treatment. Most patients saw their health improve during hospitalisation (Karnofsky index raise from 70% before treatment to 86% after treatment and general health state improvement of 85% of the patients as judged by the Investigators, data not shown). Concomitant treatments used to manage adverse events and co-existing diseases were mainly antiparasitic, antiinfective, analgesic, antipyretic, antiemetic drugs and benzodiazepines.

### Limitations

The evaluation of adverse events and causes of mortality was complex, as symptoms were often related and confused with the symptoms of the disease itself [23], [24] or concomitant disease in a background of severely ill and often malnourished patients. Apart from nausea and vomiting, the symptoms observed during NECT treatment are similar to HAT symptoms described during anamnesis at enrolment and reported in the literature [23]. A clear distinction of causality between the disease itself, the co-morbidities and the study treatment is not possible in most cases.

Similar in-hospital safety rates and profiles were observed among the different population sub-groups including the small children, pregnant (considering the low number in this group) or breastfeeding women and patients having previously relapsed.

Implementing a clinical trial inevitably modifies field conditions, as study procedures, patient's safety recommendations, study forms and external expertise are being brought to the centres. Nevertheless, the study was carefully designed to minimise these modifying effects, while maintaining adequate quality standards. The facilities' hospitalisation conditions were maintained: for example food was not systematically provided, unless a patient was suffering from advanced malnutrition; concomitant medication was based on routine stock of drugs. However, for safety reasons, emergency kits containing drugs for treatment of potentially severe or life threatening events were provided to the centres before the inclusion of the first patient. The study was implemented in six treatment facilities in the DRC under conditions mimicking field reality as much as possible.

### Comparison to previous studies and generalisability

The demographic characteristics of patient populations varied slightly between facilities, but nonetheless reflected the observed distribution of the HAT populations described in the literature [4], [13] and therefore allows comparison with previous studies even if here additional groups have been added with similar baseline and in-hospitalisation safety profiles.

The proportion of patients discharged alive after NECT treatment in field conditions was comparable to eflornithine monotherapy and to previous NECT trials in field conditions, varying from 94.1% to 99.3%, depending on the drug in use [4], [11], [13], [14], [21], [22]. The fatality rate during hospitalisation did not exceed the projected values derived from literature and field reports (0.8–2.1%; [4], [11], [13], [14]).

The median length of hospitalisation for a patient was 16 days including premedication and observation period compared to 25–30 days for melarsoprol [3] or 20 days for eflornithine monotherapy (personal communication PNLTHA DRC). The NECT therapy could be accomplished in 10 days for most patients. Its dosage schedule is simpler to apply than eflornithine monotherapy (56 versus 14 intravenous infusions [4]), requiring less nursing staff.

Compared to the formerly used standard HAT treatments, NECT is safer than melarsoprol (2.7–34% melarsoprol case fatality rate [3], [4]) and similar to eflornithine monotherapy.

The overall safety profile of NECT in the present study is similar to the safety data of NECT obtained during previous studies comparing different drug combinations [21], Nifurtimox-Eflornithine case series [22] and the NECT phase III RCT comparing NECT to eflornithine monotherapy [11]. In this study, arrhythmias, musculoskeletal and connective tissue disorders, injection site reactions and headaches were reported less frequently compared to the NECT phase III RCT [11]. All other adverse events were similar in nature, intensity and frequency. These differences in adverse event reporting can be explained by the different design and context of both studies. Another limitation of the comparability is that adverse events reported during previous studies were coded according to different standard dictionaries (i.e. the CTC).

Potentially harmful neurological adverse events, such as convulsions (9%) and coma (1%) compatible with an encephalopathic syndrome were observed in 13 SAE cases (2.1%). However, as convulsions already occurred in 31 patients (5%) prior to treatment (Table 1), it is difficult to assess if they were caused by the disease, were related to the treatment regimen or resulted from a combination of both. These cases of encephalopathic-like reactions are not directly comparable to previous eflornithine [4], [13], [14] or melarsoprol [3], [25], [26] trials, as the same definition of the encephalopathic syndrome [25], [27] was not consistently used in all those trials.

Switching from a controlled environment, such as during the NECT phase III RCT where patients were closely followed [11] to field conditions raised some concerns about infection risk at the injection site and infections in general. However, during the present trial, no evidence of increased infection was observed.

As already expressed by Priotto [11], vomiting remains a concern because of its frequency, especially during the first days of NECT therapy. Nifurtimox doses were repeated once or twice in 6.5% of the patients. The final effectiveness analysis will enable evaluation of the impact of vomiting on the cure rate. A close observation of nausea and vomiting is recommended in order to allow the timely administration of a second nifurtimox dose if necessary, as well as the prescription of an anti-emetic drug.

### Conclusions and recommendations

The in-hospital safety and feasibility of NECT in field conditions have been shown to be satisfactory in this trial. Consequently, its use in remote, rural sleeping sickness treatment facilities in endemic countries seems justified. In field conditions and in a wider population, including children, the use of NECT displayed a similar tolerability profile to that previously described in more stringent clinical trial conditions [11]. However, wide implementation of NECT demands sufficient levels of human resources for IV infusion treatment and that financial and logistical barriers in the supply chain management are overcome. In addition, a common international, standardised pharmacovigilance system should be supported to further improve the collection of safety and efficacy data in specific populations (pregnant/breastfeeding women and children) and to monitor the emergence of rare severe adverse events.

The in-hospital evolution of patients was similar to previously published NECT results. The effectiveness will be assessed at the end of the 24 months follow-up period.
